# The incidence, characteristics, management and outcomes of anaphylaxis in pregnancy: a population‐based descriptive study

**DOI:** 10.1111/1471-0528.15041

**Published:** 2018-01-03

**Authors:** SJ McCall, KJ Bunch, P Brocklehurst, R D'Arcy, K Hinshaw, JJ Kurinczuk, DN Lucas, B Stenson, DJ Tuffnell, M Knight

**Affiliations:** ^1^ Policy Research Unit in Maternal Health and Care National Perinatal Epidemiology Unit (NPEU) Nuffield Department of Population Health University of Oxford Oxford UK; ^2^ Birmingham Clinical Trials Unit University of Birmingham Birmingham UK; ^3^ Nuffield Department of Obstetrics and Gynaecology John Radcliffe Hospital University of Oxford Oxford UK; ^4^ Department of Obstetrics and Gynaecology Sunderland Royal Hospital Sunderland UK; ^5^ Department of Anaesthetics Northwick Park Hospital London UK; ^6^ Neonatal Unit Royal Infirmary of Edinburgh Edinburgh UK; ^7^ Teaching Hospitals Foundation NHS Trust Bradford UK

**Keywords:** Anaphylaxis in pregnancy, causal agents, management, outcomes

## Abstract

**Objective:**

The aim of this study was to estimate the incidence of anaphylaxis in pregnancy and describe the management and outcomes in the UK.

**Design:**

A population‐based descriptive study using the UK Obstetric Surveillance System (UKOSS).

**Setting:**

All consultant‐led maternity units in the UK.

**Population:**

All pregnant women who had anaphylaxis between 1 October 2012 and 30 September 2015. Anaphylaxis was defined as a severe, life‐threatening generalised or systemic hypersensitivity reaction.

**Methods:**

Prospective case notification using UKOSS.

**Main outcome measures:**

Maternal mortality, severe maternal morbidity, neonatal mortality and severe neonatal morbidity.

**Results:**

There were 37 confirmed cases of anaphylaxis in pregnancy, giving an estimated incidence of 1.6 (95% CI: 1.1–2.2) per 100 000 maternities. Four cases of anaphylaxis were in women with known penicillin allergies: two received co‐amoxiclav and two cephalosporins. Twelve women had anaphylaxis following prophylactic use of antibiotics at the time of a caesarean delivery. Prophylactic use of antibiotics for Group B streptococcal infection accounted for anaphylaxis in one woman. Two women died (5%), 14 (38%) women were admitted to intensive care and seven women (19%) had one or more additional severe maternal morbidities, which included three haemorrhagic events, two cardiac arrests, one thrombotic event and one pneumonia. No infants died; however, in those infants whose mother had anaphylaxis before delivery (*n* = 18) there were seven (41%) neonatal intensive care unit admissions, three preterm births and one baby was cooled for neonatal encephalopathy.

**Conclusions:**

Anaphylaxis is a rare severe complication of pregnancy and frequently the result of a reaction to antibiotic administration. This study highlights the seriousness of the outcomes of this condition for the mother. The low incidence is reassuring given the large proportion of the pregnant population that receive prophylactic antibiotics during delivery.

**Tweetable abstract:**

Anaphylaxis is a rare severe complication of pregnancy and frequently the result of a reaction to antibiotic administration.

## Tweetable abstract

Anaphylaxis is a rare severe complication of pregnancy and frequently the result of a reaction to antibiotic administration.

## Introduction

Anaphylaxis in pregnancy is a potentially fatal systemic hypersensitive reaction, which is rapid in onset. It is characterised by life‐threatening airway, breathing or circulatory problems, often with skin or mucosal change. The aetiology of anaphylaxis in pregnancy includes exposure to allergens such as antibiotics and latex.^1^, ^2^, ^3^, ^4^, ^5^ It commonly occurs when allergens trigger an IgE mechanism causing mast cell activation, resulting in an anaphylactic reaction.^6^

There is very little information regarding the incidence of anaphylaxis in pregnancy in the UK. A study in the USA suggested an incidence of 2.7 cases per 100 000 deliveries.^7^ It has been proposed that anaphylaxis is increasing in the general population.^8^, ^9^ This is of particular concern as case reports indicate that maternal and neonatal outcomes of anaphylaxis in pregnancy are severe.^10^, ^11^ Complications include cardio‐respiratory compromise for both the woman and the infant, and hypoxic brain injury in the neonate. However, these case reports are unlikely to be representative of all women with anaphylaxis in pregnancy.

Exposure to antibiotics is increasing in the pregnant population through the use of prophylactic antibiotics before elective caesarean delivery, and intrapartum antibiotic prophylaxis for group B streptococcal (GBS) carriage to prevent neonatal transmission,^1^, ^2^ although routine screening for GBS carriage is not recommended in the UK.^12^, ^13^

There are no specific guidelines for the management of maternal anaphylaxis so it is likely that women with anaphylaxis in pregnancy are treated in the same way as those in the general population.^14^, ^15^ Management is characterised by a rapid assessment of life‐threatening cardio‐respiratory compromise, which is usually treated with epinephrine, hydrocortisone, chlorphenamine, oxygen and fluids.^16^ However, there has been no population‐based study to describe whether this management is used in practice.

### Study objective

This study identified a population‐based cohort of women who had anaphylaxis in pregnancy in order to describe the incidence, causative agents, management and associated outcomes of this condition.

## Methods

### Case definition

Anaphylaxis is defined as a severe, life‐threatening generalised or systemic hypersensitivity reaction. Cases had to have at least one of the following criteria: a life‐threatening airway, breathing or circulatory problem (defined in Box 1). The condition must have been of sudden onset with rapid progression of symptoms, with skin and/or mucosal changes. However, during the study it became evident that skin or mucosal changes were not evident if the management was rapid. Therefore, all women in whom the final clinical diagnosis was anaphylaxis and who met the criteria in Box 1, irrespective of the presence or absence of skin/mucosal changes, were included**.**

Box 1 **Definitions of clinical criteria**

The presence of at least one of the following:

1 A life‐threatening airway problem is taken to include:
  - Laryngeal or pharyngeal oedema
  - Hoarse voice
  - Stridor
2 A life‐threatening breathing problem is taken to include:
  - Shortness of breath and raised respiratory rate
  - Wheeze
  - Decreased oxygen saturations
  - Confusion secondary to hypoxia
  - Cyanosis
  - Respiratory exhaustion or respiratory arrest
3 A life‐threatening circulatory problem is taken to include:
  - Signs of shock such as faintness, pallor or clammy skin
  - Tachycardia >100 bpm
  - Systolic BP <90 mmHg
  - Decreasing level of consciousness
  - Signs of ischaemia on ECG
  - Cardiac arrest

### Data source

This population‐based descriptive study prospectively collected cases of anaphylaxis in pregnancy from all obstetrician‐led maternity units in the UK using the UK Obstetric Surveillance System (UKOSS) between 1 October 2012 and 30 September 2015. The UKOSS methodology has been described elsewhere.^17^ In brief, a monthly mailing card was sent to nominated clinicians in each consultant‐led obstetric unit; this card had a simple tick box to signify whether there had been a case of anaphylaxis in pregnancy that month in the unit. Clinicians also returned cards if there had been no cases of anaphylaxis in pregnancy. The ‘nil to report’ system allowed the differentiation between a non‐response and those where there were truly no cases, which allowed further follow up of non‐responders. Once a case was reported, the reporting clinician was sent a data collection form to collect information on demographic characteristics, obstetric history, causative agents, management and outcomes for each case. No identifiable information was received and the data were double‐entered onto a bespoke database. Ethics approval for the anonymised UKOSS data collection was granted by the London Multi‐centre Research Ethics Committee (04/MRE02/45).

This study aimed to assess the incidence of anaphylaxis in pregnancy in the UK, therefore the study power was governed by the number of cases occurring. All analyses were completed using STATA (v13SE, College Station, TX, USA). The incidence rate with a 95% CI was calculated using 3 years of total maternities in the UK (2012–2014). The denominator data were obtained from the Office for National Statistics for England and Wales, NHS digital and the National Records of Scotland. The mode of delivery denominator data were obtained from the Hospital Episode Statistics Analysis, Health and Social Care Information Centre for England, Patient Episode Database for Wales via the Welsh Government, Information Service Division for Scotland and Northern Ireland Statistics and Research Agency for 2012–2013, which was extrapolated for the subsequent years. The results were presented with absolute values and proportions to describe the characteristics, suspected causative agents, management and outcomes of the cases. Timing from symptoms of anaphylaxis to delivery was used to categorise women into groups according to when the reaction occurred in relation to the delivery. These were: antepartum, intrapartum (immediately before delivery), post‐delivery (immediately after delivery) and late postpartum.

## Results

During 2012–2015, there were 46 case notifications; of these, 37 women met the case definition. In an estimated 2 324 552 maternities, there was thus an estimated incidence of 1.6 (95% CI: 1.1–2.2) cases per 100 000 maternities.

Table 1 shows the characteristics and management of women with anaphylaxis in pregnancy. The mean age of women was 33 (±7 SD) years. Twenty (54%) women had a history of previous allergic reactions, 16 (43%) with a history of atopy and two (5%) with a previous anaphylactic reaction. [Correction added on 27 February 2018, after first online publication: ‘Twenty‐nine (78%)’ has been changed to ‘Twenty (54%)’ in the preceding sentence.] Approximately a third of women (*n* = 12) had a known drug allergy and the majority of these reported allergies related to penicillin‐based drugs (*n* = 10).

**Table 1:** Characteristics and management of women with anaphylaxis during pregnancy in the UK between October 2012 and September 2015 (*n* = 37)

Characteristics	*n* (%)
**Mean age (SD), ** ***n*** ** = 36**	33 (7)
**Ethnic group**	
White	28 (76)
Non‐White	9 (24)
**Smoking status**	
Never/ex‐smoker	29 (78)
Smoked during pregnancy	8 (22)
**Median gestational age at delivery (IQR)** a **, ** ***n*** ** = 34**	39 (37–40)
**Median BMI (IQR), ** ***n*** ** = 35**	26 (23–30)
**Previous obstetric history**	
Parity	
0	14 (38)
1 or more	23 (62)
Previous pregnancy problem	
Yes	14 (38)
**Previous medical history**	
Previous anaphylactic reaction	2 (5)
Known drug allergy	12 (32)
Type of drug allergy^a^	
Penicillin based	10 (83)
Other	2 (17)
Have a history of allergic reactions	20 (54)
History of atopy	16 (43)
**Management** a	
High flow O_2_	29 (81)
IV fluids	32 (86)
Epinephrine	28 (93)
Chlorphenamine	28 (88)
Hydrocortisone	33 (97)
Tryptase levels tested	
Yes	31 (84)
If yes, Had abnormal tryptase levels	9 (29)

a Percentages are calculated for those with complete data.

[Correction added on 27 February 2018, after first online publication: In table 1, the *n* (%) values for ‘have a history of allergic reactions’ have been changed to ‘20 (54)’ from ‘29 (78)’.]

The management of the women is presented in Table 1. High flow oxygen was administered to 29 (81%) women and intravenous fluids to 32 (86%) women. The majority of women received epinephrine (*n* = 28, 93%), chlorphenamine (*n* = 28, 88%) and/or hydrocortisone (*n* = 33, 97%). Tryptase levels were measured in 31 (84%) women after resuscitation and were raised in nine (29%).

**Figure 1:**
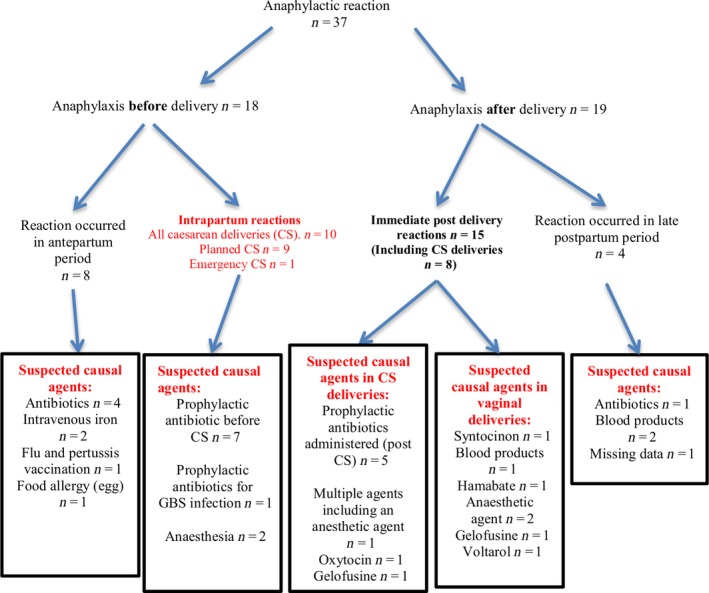
Clinical history of anaphylaxis and suspected causal agent according to time of reaction.

Women with anaphylaxis in pregnancy were divided into four groups according to the timing of the reaction (Figure 1). The majority of reactions occurred intrapartum (*n* = 10) or post‐delivery (*n* = 15). There were fewer reactions in the antepartum period (*n* = 8) and the postpartum period (*n* = 4).

For those who had anaphylaxis symptoms in the intrapartum period, the median time to delivery was 15 minutes after symptoms of anaphylaxis were first observed (IQR: 6–18). For those who had a caesarean section, the median time to delivery after a reaction to a prophylactic antibiotic was 6 minutes (IQR: 4–34). For those who had anaphylaxis symptoms immediately post‐delivery, the median time to symptoms of anaphylaxis after delivery was 25 minutes (IQR: 9–64).

In the intrapartum group, seven women had a reaction to prophylactic antibiotics before a caesarean delivery and two reacted to anaesthesia (Figure 1). One woman had a reaction to antibiotics given for GBS prophylaxis and then proceeded to an immediate caesarean delivery. Nine caesarean deliveries were already planned and there was only one emergency caesarean delivery as a result of anaphylaxis. In the post‐delivery group, five of these women had a reaction to a prophylactic antibiotics after a caesarean delivery. In total, 12 women had a reaction to prophylactic antibiotics given at the time of caesarean delivery. The incidence of prophylactic antibiotic‐related anaphylaxis associated with caesarean delivery was 2.1 (95% CI: 1.1–3.6) per 100 000 caesarean deliveries. Overall, half of the women had a suspected reaction to an antibiotic across all timing groups (*n* = 18, 49%). The agents responsible in the anaesthetic reactions were: suxamethonium, thiopentone and a component of spinal anaesthesia.

The anaphylactic reaction was characterised by the majority of women having either pulmonary or circulatory symptoms; 24 (65%) additionally had skin or mucosal changes. The majority of the reactions occurred in the hospital setting.

The maternal outcomes for women with anaphylaxis are shown in Table 2. Two women died (case fatality 5%, 95% CI: 0.7–18.2%), seven women had one or more additional severe maternal morbidities, which included three haemorrhagic events, two cardiac arrests, one pulmonary embolism (PE) and one woman had pneumonia. The majority of poor maternal outcomes occurred in the post‐delivery group (two maternal deaths, one cardiac arrest, three haemorrhagic events and PE). The agents responsible for the reactions in the two women who died were suxamethonium and co‐amoxiclav.

**Table 2:** Maternal outcomes in women who had anaphylaxis during pregnancy

Maternal outcomes	Reaction before delivery, *n* = 18 (%)	Reaction after delivery, *n* = 19 (%)	Total number = 37, *n *(%)
**Maternal death**	0	2 (11)	2 (5)
**Severe maternal morbidity** a	2 (11)	5 (26)	7 (19)
Cardiac arrest	1 (6)	1 (5)	2 (5)
Haemorrhage	–	3 (16)	3 (8)
Thrombotic event	–	1 (5)	1 (3)
Pneumonia	1 (6)	–	1 (3)
**ITU admission**	5 (28)	9 (47)	14 (38)

a Includes the morbidities of the women who died.

Two women had a pregnancy loss prior to the 3rd trimester and there was one case without delivery information, thus delivery data were available for 34 singleton pregnancies. The infant outcomes are presented in Table 3. There were no stillbirths. No infants died; however, in those infants whose mother had anaphylaxis before delivery there were seven (41%) neonatal intensive care unit admissions, three preterm deliveries and one baby had whole body cooling for neonatal encephalopathy.

**Table 3:** Infant outcomes in women who had anaphylaxis during pregnancy (*n* = 17^a^)

Neonatal outcomes	Total no. before delivery, *n* (%)	Group with prophylactic use of antibodies prior to caesarean section (*n* = 7), *n* (%)
Neonatal ICU admission	7 (41)	5 (71)
Neonatal encephalopathy	1 (6)	0
Apgar at 5 minutes <7	0	0
Resuscitation needed for infant	6 (35)	6 (86)

a One infant had no known delivery information.

## Discussion

### Main findings

This study presents national prospective data collected on women with anaphylaxis in pregnancy. Anaphylaxis is a severe condition in pregnancy and this study has shown that it is frequently the result of a reaction to antibiotic administration. A third of the reactions were as a result of the prophylactic use of antibiotics at the time of a caesarean section. In total, half of the reactions were due to an antibiotic. This study highlights the seriousness of the outcomes of this condition for both women and infants born as a result of exposure to anaphylaxis. Nevertheless, the low incidence is reassuring given recent changes in antibiotic prophylaxis policies.^18^ To our knowledge this is the first national prospective population‐based study on anaphylaxis in pregnancy and provides population‐based data on the causal agents and outcomes of this severe but rare condition.

### Strength and limitations

The main strength of this study is the use of the UKOSS methodology, which has provided prospectively collected population‐based data on women with anaphylaxis in pregnancy. The adoption of a strict case definition has prevented the inclusion of false‐positive cases. However, it is possible that less severe cases of anaphylaxis were not reported and the incidence of anaphylaxis may be underestimated. A possible limitation is that only 37 cases were identified; this has resulted in wide confidence intervals in estimations of incidence and the case fatality rate, even in this national population‐based study.

### Interpretation

#### Incidence

The incidence of anaphylaxis in pregnancy in this study at 1.6 (95% CI: 1.1–2.2) per 100 000 maternities is similar to that of previously published studies. A study from Texas using an obstetric database estimated the incidence to be 2.7 per 100 000 deliveries (95% CI: 1.7–4.2).^7^ Furthermore in 2003–2005, a study of severe maternal morbidity in Scotland using data collected from consultant‐led units, estimated the incidence as three cases per 100 000 maternities (95% CI: 1–7).^19^ In the USA there is routine bacteriological screening for GBS, which has increased the proportion of women receiving intrapartum antibiotics for GBS; this is may explain the apparent higher incidence in the Texan study.^20^ Other potential reasons include false‐positives in the Scottish and Texan studies.

During 2012–2014, the maternal mortality rate was 8.54 per 100 000 maternities (95% CI: 7.40–9.81).^21^ In comparison, the maternal mortality rate as a result of anaphylaxis was 0.09 per 100 000 maternities (95% CI: 0.01–0.30) over a similar time period.

#### Causal agents

Twelve reactions were the direct result of prophylactic use of antibiotics at the time of caesarean delivery which were administered according to current guidelines.^18^ Five of these reactions occurred after caesarean delivery, which is not a currently recommended practice. This is important; had the antibiotics been administered before surgery, the burden of infant morbidity might have been higher.

Similar to previous studies, the most common suspected causal agent was the use of antibiotics.^7^, ^22^ Previous studies showed women developed anaphylaxis after being given antibiotics prophylactically for GBS,^7^, ^23^, ^24^ whereas others were given for surgical prophylaxis.^22^ Only one woman developed anaphylaxis following antibiotics for prophylaxis of GBS in the UK, which may reflect national differences in antenatal screening policies for GBS between the UK and some other countries.^12^, ^25^ Despite both penicillin and cephalosporin drugs being the most common triggers of a reaction, they were commonly used in this population. There is still debate about the optimal choice of antibiotic for caesarean section prophylaxis.^26^

Two women who had known penicillin allergies were given a penicillin‐based antibiotic, resulting in an anaphylactic reaction. Human factors have been demonstrated to play a role in medication errors.^27^ This highlights that these cases were preventable and indicates that a detailed drug allergy history must be taken at booking or prior to administration of any antibiotics and communicated to the clinical team.

#### Management

The time from detection of first symptoms of anaphylaxis to diagnosis was short and mucosal and skin changes did not occur in 35% of this cohort, which suggests that prompt management taken by the clinical team prevented symptoms from progressing. This would suggest that skin and mucosal changes should not be part of the main case definition of anaphylaxis.

The causal relationship between anaphylactic reactions and caesarean delivery has been unclear. Only one woman had a caesarean delivery as the result of an anaphylactic reaction; this woman received antibiotics for GBS prophylaxis and then went on to be delivered via caesarean section. Although the other causal agents (anaesthetics and prophylactic use of antibiotics before a caesarean delivery) were given before a planned caesarean, the women concerned went on to have a reaction. Previous studies have shown that most women with anaphylaxis were delivered by caesarean section.^7^, ^22^ A previous review has recommended caesarean delivery in severe cases of anaphylaxis.^22^ Caesarean delivery in this scenario would potentially reduce the risk of hypoxic injury in the infant if the woman's circulatory system were compromised due to a severe anaphylactic reaction.

The majority of patients were treated according to the normal anaphylaxis algorithm as they received high level oxygen, epinephrine, hydrocortisone and chlorphenamine.^14^ However, national adherence to national guidelines could be improved, as only 81% received oxygen and 87% received IV fluids. Consistent with current guidelines,^14^ our study results show almost all cases with a recorded response received epinephrine. In pregnancy it has been suggested that epinephrine may inhibit utero‐placental blood flow.^28^ However, fetal survival is maximised through maternal resuscitation.^29^ Findings from the Confidential Enquiry into Maternal Death in the UK recommended that epinephrine should be the first line treatment for anaphylaxis in pregnancy.^22^, ^30^ In addition, to aid clinical expedience, the anaphylaxis algorithm should be visible in operating theatres and labour wards.^14^, ^15^

#### Outcomes

Previous studies have been unable accurately to examine maternal and infant outcomes in this condition due either to publication bias in case reports^22^ or to lack of suitable population‐based data.^7^ This study has shown there are poor outcomes for women who have anaphylaxis with a 5% case fatality rate and a fifth of women having a severe morbidity. The confidential enquiry into maternal deaths in the UK would suggest a similar case fatality rate for anaphylaxis during pregnancy, with one death due to anaphylaxis during 2006–2008.^30^

Unlike previous research,^28^ outcomes were good for the majority of infants; however, there was one case of neonatal encephalopathy which would have resulted in whole body cooling. Previous research has suggested that poor outcomes have been averted by rapid diagnosis of anaphylaxis, appropriate administration of epinephrine, and prompt delivery after diagnosis (within 10–15 minutes).^22^ It would appear that management of maternal anaphylaxis in this study might explain the low prevalence of poor neonatal outcomes, noting, however, the wide confidence intervals around the estimate of neonatal encephalopathy incidence due to the low numbers.

## Conclusions

Anaphylaxis is a rare condition in pregnancy in the UK and is often the result of antibiotic administration with severe adverse outcomes for both mother and child. Confirmation of the causative agent in all women with a reaction should be undertaken to prevent the prospect of future anaphylactic reactions. Further efforts are required to ensure that women with known allergies are not given drugs they are sensitised to.

### Disclosure of interests

Full disclosure of interests form available to view online as supporting Information.

### Contribution to authorship

PB and MK conceived the study. MK, RD, BS, PB, NL, DJT, KH and JJK contributed to study design. RD designed the data collection form. MK, SJM and KJB analysed the data. SJM wrote the first draft of the manuscript. All authors interpreted the data and edited the manuscript.

### Details of ethics approval

Ethics approval for the anonymised UKOSS data collection was granted by the London Multi‐centre Research Ethics Committee (04/MRE02/45).

### Funding

This paper reports on an independent study which is part‐funded by the Policy Research Programme in the Department of Health. MK is funded by an NIHR Research Professorship. SM is funded by the Nuffield Department of Population Health and Medical Research Council (MRC) training grant MR/K501256/1. The views expressed in this publication are those of the author (s) and not necessarily those of the MRC, the NIHR or the Department of Health. The funders had no role in the study design, data collection and analysis, decision to publish or preparation of the article.
